# Tethered from the Head and from the Tail: The Structure of Hydroxyl-Functionalized Ionic Liquids

**DOI:** 10.1021/acs.jpclett.5c03046

**Published:** 2025-12-11

**Authors:** Raphael Ogbodo, Ieesha Ansar, Clinton Adu, Sharon I. Lall-Ramnarine, James F. Wishart, Andrew J. Nieuwkoop, Claudio J. Margulis

**Affiliations:** † Department of Chemistry, 4083 The University of Iowa, Iowa City, Iowa 52242, United States; ‡ Department of Chemistry, 14782 Queensborough Community College-CUNY, Bayside, New York 11364, United States; ¶ Department of Chemistry and Chemical Biology, Rutgers University, Piscataway, New Jersey 08854, United States; § Chemistry Division, 8099 Brookhaven National Laboratory, Upton, New York 11973-5000, United States

## Abstract

Ionic liquids with special functionalities are synthesized with the specific purpose of creating new patterns of interaction in the condensed phase. This Letter discusses the case of alcohol-functionalized ILs, the so-called HFILs, which are part of the larger cohort of task-specific ionic liquids. We find that this small chemical modification can cause massive changes in the liquid landscape when the cationic tails are longer. For prototypical ionic liquids, larger alkyl tails act as separators of charge networks, but in the case of HFILs these become physical charge network linkers. The OH functionality adds a large repertoire of interactions and correlations that were mostly unavailable to traditional ILs.

## Graphical abstract

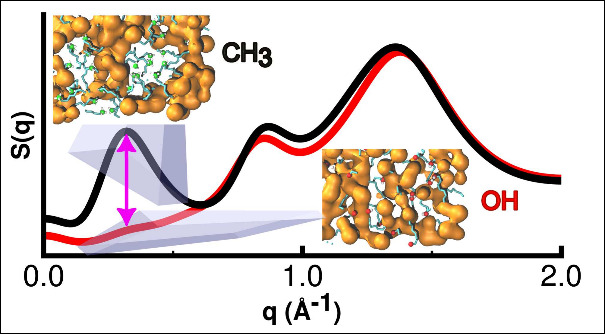

Functionalized ionic liquids (ILs), also referred to as task-specific ILs (TSILs),^1^ are designer ILs created with particular applications in mind; popular among these are gas capture, separations, and catalysis. This article focuses on a special class of TSILs, the so-called hydroxy-functionalized ILs (HFILs)^2–8^ shown in Figure 1a, all of which have a terminal OH group in the longer cationic alkyl tail. We study these ${{\mathrm{NTf}}_{2}}^{-}$ and FSI^–^-based HFILs experimentally and computationally in comparison with their nonfunctionalized analogues depicted in the same figure. HFILs have shown potential for a variety of applications including the selective extraction of metal ions as well as other molecular compounds.^9–11^ For HFILs in Figure 1a, details of their synthesis, their physical characterization, the determination of their physical properties, and the computational methodology used to study them are all presented in Section S.1. As can be gleaned from Figure 1b, the match between computationally determined and experimentally measured densities for HFILs and their IL counterparts is excellent.

**Figure 1:**
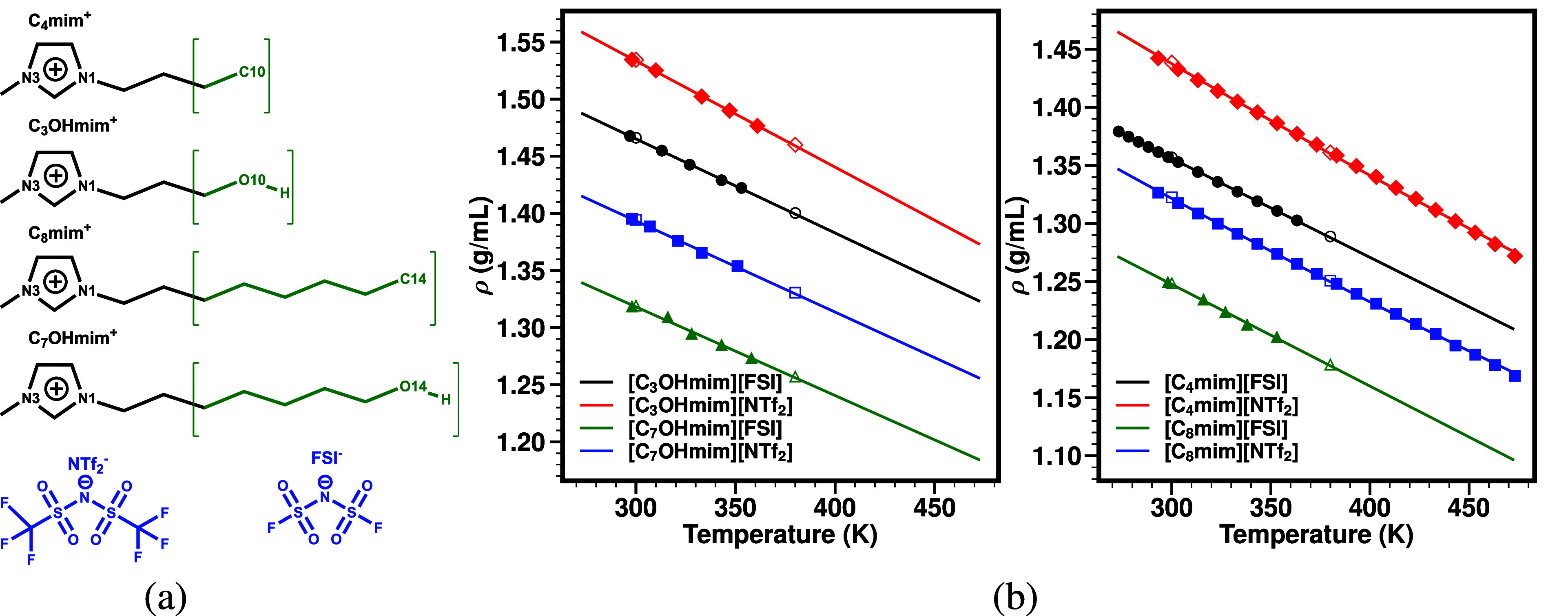
(a) Ions and specific atoms within them labeled or color-coded to match definitions for anion, cationic head, and tail used for structure function (*S*(*q*)) calculations; (b) temperature-dependent densities, in which filled markers represent experimentally measured points and open markers simulated values at 300 and 380 K. Lines are fits to the experimental values (see Tables S.1 and S.2). Fit parameters for [C_4_mim]­[FSI] are from ref 12, whereas those for [C_4_mim]­[NTf_2_] and [C_8_mim]­[NTf_2_] are from ref 13.

HFILs present an interesting structural–dynamical interplay between charge–charge interactions that dominate the formation of charge networks and hydrogen bonding, which can be between alcohol moieties or between OH groups and the charged components of the ions. This leads to complex intra- and intermolecular patterns of interaction^4–8^ that significantly influence physicochemical properties^14–16^ when contrasted with those of otherwise similar but not hydroxylated ILs. Table 1 shows onset glass transition temperatures (*T*_g_), as well as densities, conductivities, viscosities, and Walden products (molar conductivity × viscosity) at indicated temperatures, for the set of ILs studied (for details on the techniques, see Section S.1.2). Table 1 also provides fit parameters for the logarithmic form of the Vogel–Tammann–Fulcher (VTF) equation for viscosity $\ln\left(\eta \left(T\right)\right)=\ln\left(\eta_{0}\right)+\frac{DT_{0}}{T-T_{0}}$ with a data point of 1 × 10^13^ cP included at *T*_g_ for each IL,^17^ as described in ref 18.

**Table 1:** Physical Properties and Viscosity VTF Fitting Parameters for ILs and Their HFIL Analogues

Ionic Liquid	*T*_g_ (K)	Density (g/mL) @ 298 K	Conductivity (mS/cm) @ 298 K	Viscosity (cP) @ 298 K	Walden product (P S cm/mol) @ 298 K	ln(η_0_/cP)	*D*	*T*_0_ (K)
[C_3_OHmim][FSI]	193​^b^	1.47 at 297 K	4.14	65.​4^a^	0.59	–1.78	4.58	168.5
[C_4_mim][FSI]	172​^20^	1.35 ^12^	8.16 ^12^	32.​9 ^12^	0.64	–1.92^f^	5.64^f^	146.3^f^
[C_7_OHmim][FSI]	191​^c^	1.32	1.22	177​^a^	0.61	–2.41	6.66	158.8
[C_8_mim][FSI]	179​^21^	1.25	2.5 ^21^	66​^21^	0.53 ^21^	–2.32 ^21^	6.52 ^21^	149.0 ^21^
[C_3_OHmim][NTf_2_]	191​^d^	1.54	2.14	106​^a^	0.62	–2.51	6.14	160.6
[C_4_mim][NTf_2_]	186​^18^	1.440 ^18^ at 295 K	3.3 ^18^	50​^18^	0.48 ^18^	–1.93 ^18^	4.99 ^18^	160.8 ^18^
[C_7_OHmim][NTf_2_]	196​^e^	1.40	0.705	237​^a^	0.57	–2.72	6.89	161.9
[C_8_mim][NTf_2_]	187​^18^	1.28 ^18^ at 295 K	1.41 ^18^	93​^18^	0.47 ^18^	–2.44 ^18^	6.30 ^18^	156.5 ^18^

a Calculated from the VTF parameters.

b From DSC data shown in Figure S.2.

c From DSC data shown in Figure S.4.

d From DSC data shown in Figure S.1.

e From DSC data shown in Figure S.3.

f Fitted using data from refs 20 (glass transition) and^12^ (viscosities).

In the evaluation of the differences in transport properties between the HFILs and alkyl-terminated ILs, attention should first be directed at the significant differences in glass transition temperatures between the HFILs and their alkyl congeners. For the FSI^–^–ILs, the shorter- and longer-chain HFILs have glass transition temperatures that are 21 and 12 K higher than their alkyl analogues, respectively, and for the ${{\mathrm{NTf}}_{2}}^{-}$ –ILs the differences are 5 and 9 K. These increases in *T*_g_ illustrate the effect of hydroxyl-group functionalities in cross-linking the IL matrix and thereby increasing the barrier to unrestricted molecular motion connected with the glass-to-liquid transition. For all four of the HFILs, the glass transition temperatures lie in a narrow region (191 to 196 K) despite the large differences in *T*_g_ with anion type for the alkyl-terminated ILs (8–14 K), suggesting that the presence of the hydroxyl group is the most significant factor determining *T*_g_ in this case. Considering the above-discussed differences in *T*_g_, the observed viscosities and conductivities behave with temperature as expected, with the viscosities of the HFILs at 25 °C being higher than their alkyl analogues and their 25 °C conductivities being lower, in many cases by factors of about two. There is no clear trend in the fragilities of the HFIL vs alkyl ILs, as represented by the VTF *D* parameter. The 25 °C Walden products for the HFILs fall within a narrow range of 0.57 to 0.62 P S cm/mol, while those for the alkyl ILs range from 0.47 to 0.64 P S cm/mol. Based on the similarity of the two sets of Walden products it is likely that the OH-substitution does not cause any major changes in the trends of correlations and anticorrelations of translational motions of like- and unlike-charged ions that are responsible for the downward deviations from “ideal” conductivity.^19^

From an intramolecular structural perspective, we find from simulation that, except for the case of C_3_OHmim^+^, conformations of cations in HFILs and ILs are not dramatically different. This can be gleaned from Figure 2, where the cations have three peaks associated with the number of tail dihedral angles that are not in the *trans* configuration. We find that, compared to C_4_mim^+^, C_3_OHmim^+^ has a larger preference for the curled tail configuration which allows the terminal OH group to be close to the charged imidazolium ring.

**Figure 2:**
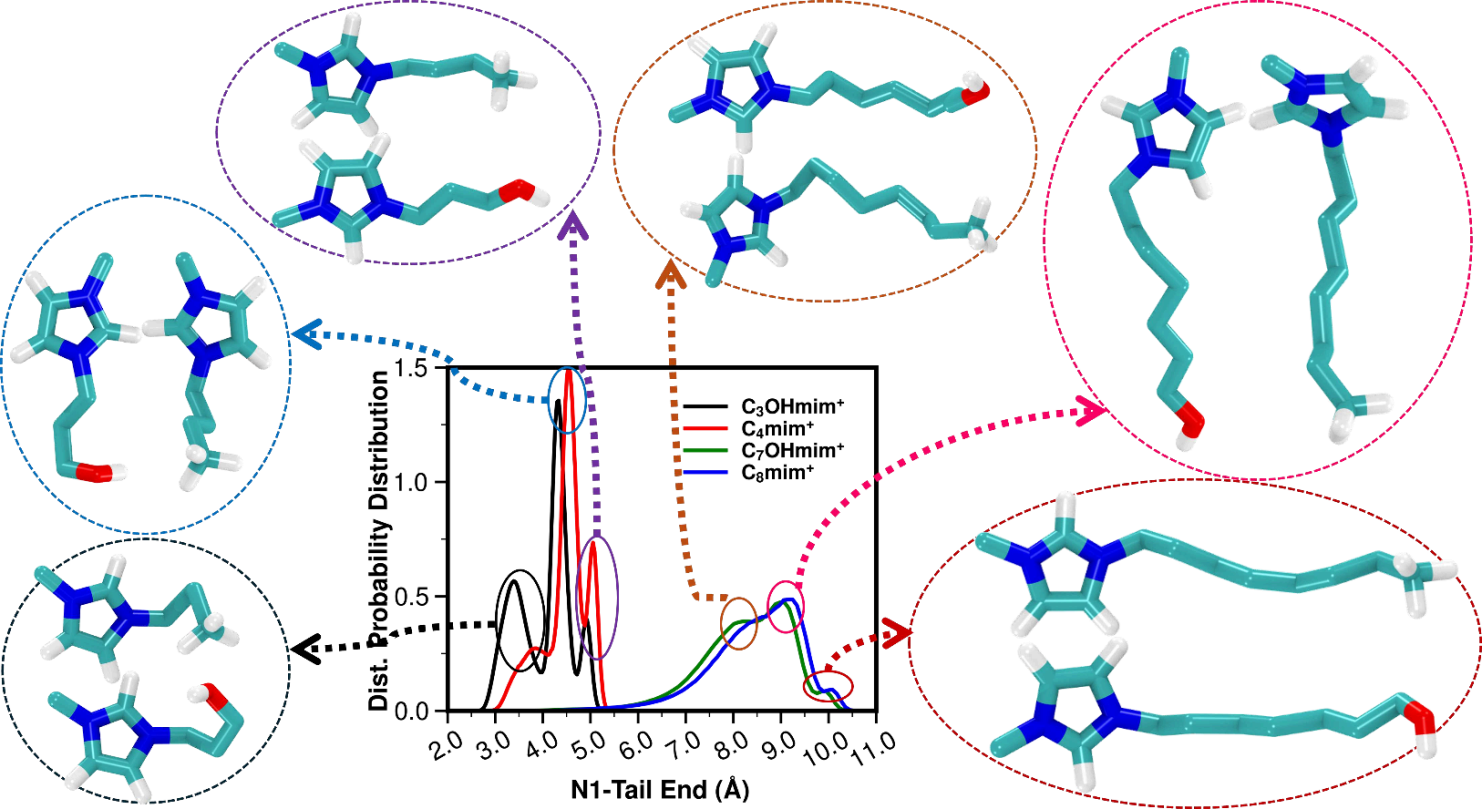
For the C_4_mim^+^, C_3_OHmim^+^, C_8_mim^+^, and C_7_OHmim^+^ cations coupled with FSI^–^, the intramolecular distance distribution between the ring nitrogen (N1) and the terminal atom of the longest side chain (C10 for C_4_mim^+^, O10 for C_3_OHmim^+^, C14 for C_8_mim^+^, and O14 for C_7_OHmim^+^). Atom nomenclature defined in Figure 1a.

As will become apparent in the discussion of Figures 3, 5, and 6 and in contrast to the deceiving similarities that are found in the intramolecular conformations of the cations shown in Figure 2, when tails are long, terminal tail OH groups may have a minor effect on intramolecular structure but majorly affect liquid structure on a global scale and, in particular, intermediate range order. OH groups increase the number of structural motifs (mostly charge alternation, and when tails are long enough, polar–apolar domain alternation) that are available to conventional ILs. If we think of prototypical ILs as being able to physically tether to form percolating charge networks, OH termination of alkyl tails creates several new ways of tethering beyond charges.^4–8^ Most important, these modes of tethering now happen at both ends of the cation, with practical implications for structure, as well as macroscopic properties including viscosity, conductivity, and density.^22–25^ Viscosities and densities are higher and conductivities lower for HFILs, and this can be gleaned from Table 1 and Figure 1b. Infrared and nuclear magnetic resonance studies show strong evidence for these new motifs,^26^ and density functional theory calculations as well as molecular dynamics (MD) studies have discussed the types of cationic aggregates that can be formed connected by hydrogen bonds; studies have also followed the dynamics of hydrogen-bonded structures.^4^

**Figure 3:**
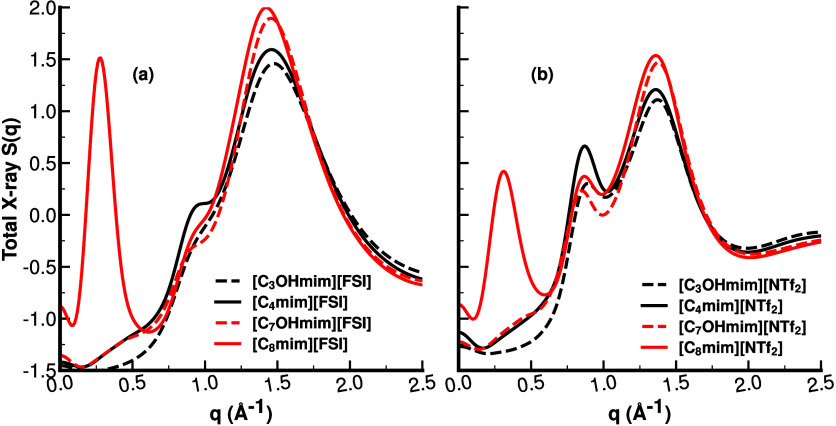
For C_8_mim^+^, C_7_OHmim^+^, C_4_mim^+^, and C_3_OHmim^+^, all coupled with FSI^–^ (left) and ${{\mathrm{NTf}}_{2}}^{-}$ (right), the total *S*(*q*) in the *q* regime between 0.0 and 2.5 Å^–1^ which includes the adjacency (∼1.4 Å^–1^), the charge alternation (*q* ∼0.85 Å^–1^) and prepeak (∼0.3 Å^–1^) regions.

When simulating each of these systems in the condensed phase, the most notable structural change derived from the structure function *S*(*q*) occurs at low *q* for the group of longer-tail liquids, those containing the C_8_mim^+^ vs C_7_OHmim^+^ cations, independent of the anion. We can glean this from Figure 3, where in each subfigure lines of the same color compare HFILs and unsubstituted ILs of similar nominal cationic lengths for a given anion.

C_8_mim^+^-containing liquids show a significant first sharp diffraction peak indicative of intermediate range order, whereas the peak at low *q* is absent in those containing C_7_OHmim^+^. Other changes can also be observed at the charge alternation region and the adjacency region but are not nearly as pronounced as in the low-*q* regime. A deeper dive into the origin of these changes requires partitioning *S*(*q*)^27–39^ into the contribution of pair subcomponents as color-coded in Figure 1a.

Figures 4 and S.5 show that HFILs containing the C_7_OHmim^+^ cation lose a majority of their tail–tail, anion–anion, cation head–cation head, and cation head–anion across-network correlations (in each case, the prepeak at low *q*) that are characteristic of nanosegregated ILs. Concomitantly, they also lose a majority of the cation head–cation tail and anion–cation tail antipeaks (anticorrelations or negative going peaks) in the same low-*q* regime. These are all conclusive evidence that, for all practical purposes, these HFILs do not have the typical tail domains responsible for intermediate range order. If we consider C_4_mim^+^-based ILs vs C_3_OHmim^+^-based HFILs, changes in the partial subcomponents of *S*(*q*) are in most cases small. Cationic head–cationic tail type correlations show some differences, particularly at lower *q* values, which is consistent with cations having different internal conformations, as depicted in Figure 2.

**Figure 4:**
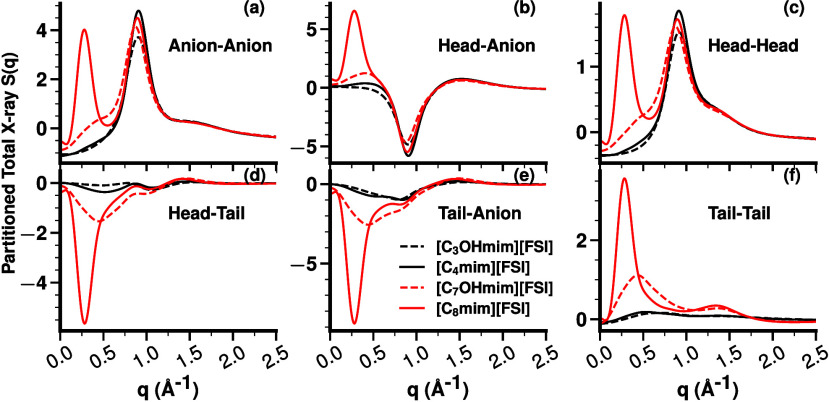
For C_8_mim^+^, C_7_OHmim^+^, C_4_mim^+^, and C_3_OHmim^+^, all coupled with FSI^–^; subionic components of the total X-ray *S*(*q*). Matching plots in the case of ${{\mathrm{NTf}}_{2}}^{-}$ are shown in Figure S.5.

What structural patterns in the liquid landscape cause the loss of tail domains and, with them, the prepeak in these HFILs? Figure 5 begins to address this from a pictorial perspective. We represent the charge network with silver surfaces; the oxygen atom in C_7_OHmim^+^ is depicted in purple when close to the charge network and in red otherwise. Consistently, the terminal tail carbon atom in C_8_mim^+^ is colored purple when close to the charge network or green otherwise. Figure 5 shows that [C_8_mim]­[FSI] has large regions that are purely tail domain where the terminal carbon in each octyl tail is depicted in green; instead, no such regions exist for [C_7_OHmim]­[FSI] where a majority of the oxygen atoms appear purple. In other words, many of the OH groups are committed to interactions with the charged portion of the ions, forming the charge network. Panel b in Figure 5 highlights with red boundary frames alcohol–alcohol hydrogen bonds. Notice that some of these include red and purple oxygen atoms implying both alcohol–alcohol and alcohol–charge interactions; the liquid landscape for ${{\mathrm{NTf}}_{2}}^{-}$ -based ILs is similar.

**Figure 5:**
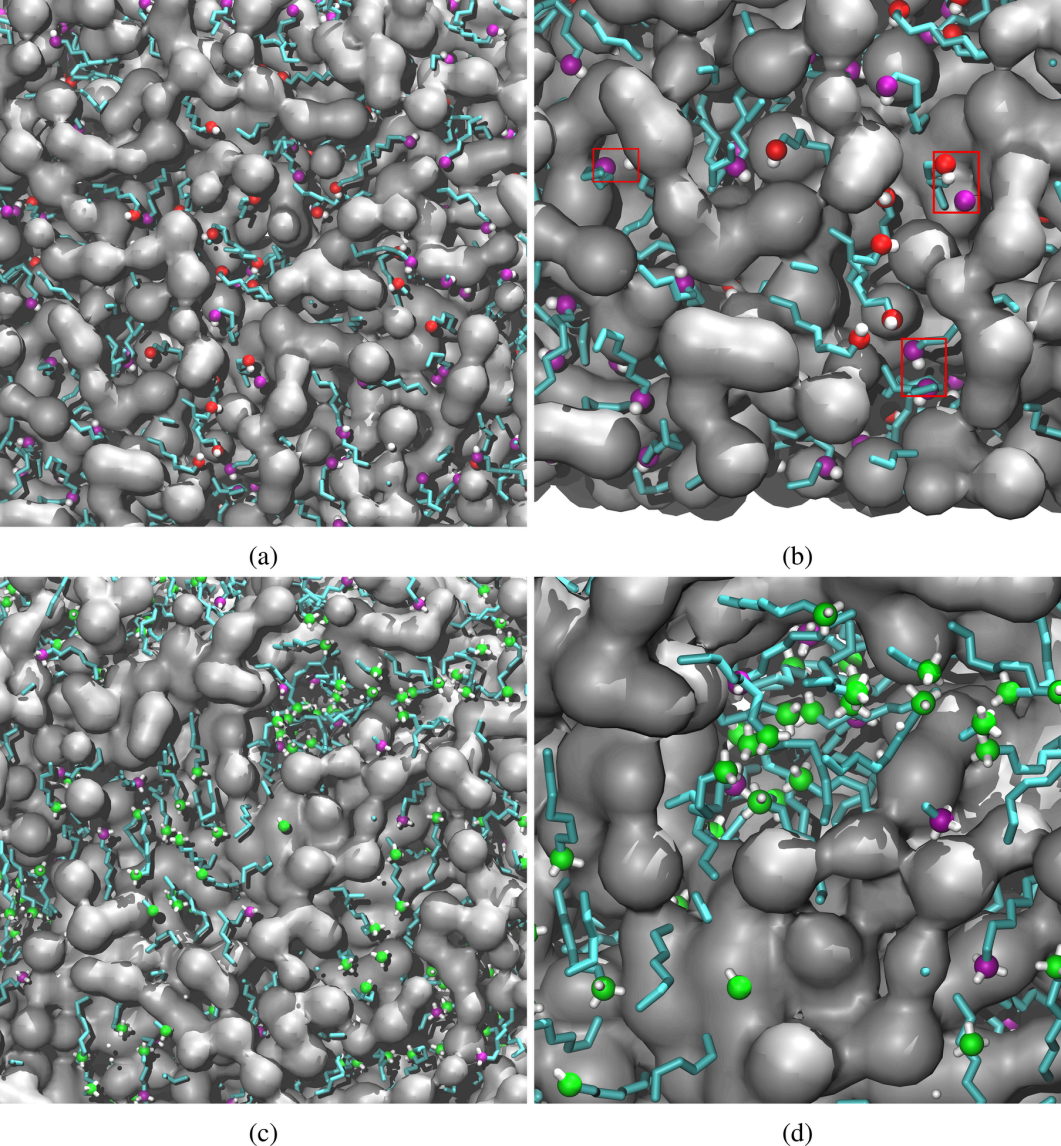
(a) Snapshot from the simulation of [C_7_OHmim]­[FSI] in which the charge network defined by virtual atomic positions corresponding to (1) the center of mass of the cationic ring including the methyl group and (2) that of the FSI^–^ anion is depicted in silver. Sites are depicted using the size of N for the cation and that of S for the anion; individual atoms in these moieties are not shown. Oxygen atoms in hydroxyl groups are depicted in red when they are more than 4.5 Å away from the virtual sites; otherwise they are depicted in purple. (b) Zoomed-in version that highlights with red frames for visual impact alcohol–alcohol hydrogen bonds. Notice that some of the oxygen atoms involved in these hydrogen bonds are depicted in red and others in purple. This means that both style interactions ion–alcohol and alcohol–alcohol are involved (see also Figure S.6). (c) Same as (a) but for [C_8_mim]­[FSI]; in this case, terminal carbon atoms further away than 4.5 Å from the network are depicted in green, whereas those closer than 4.5 Å are depicted in purple. (d) Zoomed-in version of (c); notice in (c) and (d) the clearly formed tail domains with green terminal carbon atoms; in these domains, tails from ions that are across networks do not intercalate; instead, they meet in the middle of the apolar domain.

For [C_7_OHmim]­[FSI] and [C_8_mim]­[FSI], Figure S.6 shows interionic pair distribution functions between Oan (anionic oxygen) and O14, Oan and C14, C14 and C14, and O14 and O14 (see Figure 1a for atom naming). O14–O14 shows a major peak at short distance (this corresponds to alcohol–alcohol hydrogen bonds); at about the same distance, Oan also shows a peak with alcohol O14—this is the OH groups participating in hydrogen bonds with the charge network. In contrast, there is no Oan–C14 short distance peak; this is because only the HFIL has a terminal component participating in the charge network. Consistent with depictions in Figures 5c and 5d of apolar domain aggregation for ILs of long tail that do not have terminal OH groups, C14–C14 shows a significant peak at a larger distance. For [C_8_mim]­[FSI] and [C_7_OHmim]­[FSI], Figure 6 defines the same type of interactions described earlier in Figure 5 but from a more local perspective. Figure 6a shows the characteristic pattern giving rise to intermediate range order in prototypical ILs such as [C_8_mim]­[FSI], where the charge network is seen in the middle and apolar domains flank it on the left and right. The typical distance between these tail domains separated by the charge network defines the *q* value for the prepeak. Notice that, just as in Figures 5c and 5d, alkyl groups in the tail domain do not intercalate and instead meet in the middle of the domain; this is highlighted as a cartoon-style inset in the bottom right corner of Figure 6a. Figure 6b highlights instead the prototypical pattern giving rise in *S*(*q*) to the charge alternation peak; all ILs present this ionic arrangement.

**Figure 6:**
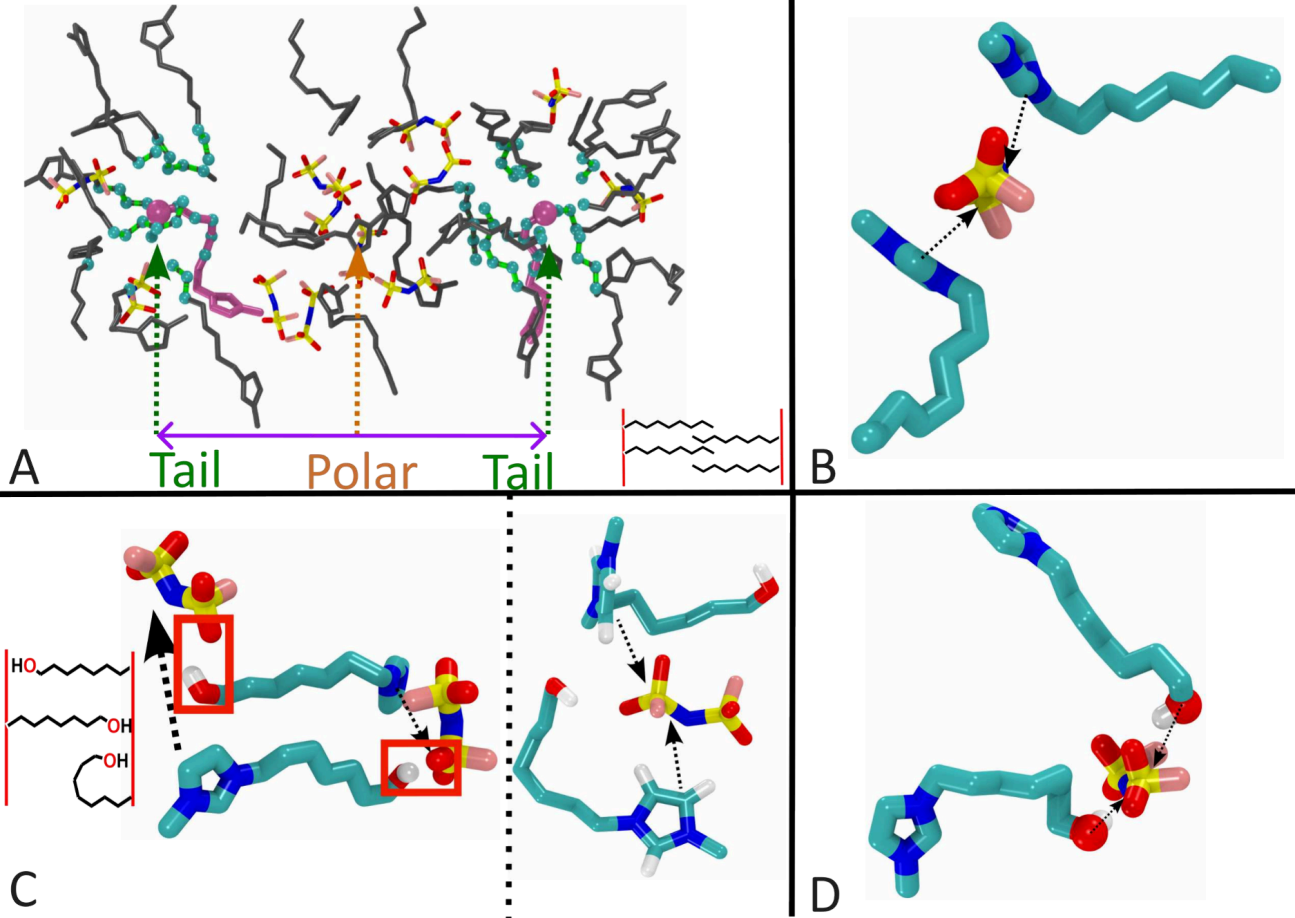
(a) For [C_8_mim]­[FSI], a typical configuration giving rise to intermediate range order and the prepreak, the purple line highlights the distance between terminal carbon atoms that belong to different apolar domains (pink cations with larger terminal carbon van der Waals radii chosen for reference) and the inset emphasizes that within an apolar domain there is no tail intercalation—in other words, tails meet in the middle of the apolar domain. (b) Typical charge alternation motif that is the basic unit of the charge network. (c) For [C_7_OHmim]­[FSI], left and right subpanels show patterns of OH tethering that we observe in a typical simulation snapshot. On the left subpanel, two charge networks are indicated with black arrows where it can be gleaned that the spacing between them is small; this is because tails do not form domains and instead reach across networks linking them. On the right subpanel, the OH group of a cation links with the oxygen atoms of a neighboring FSI^–^ counterion within the same charge network. The inset in (c) emphasizes these typical tethering patterns. (d) Similar to (b) but consisting of hydrogen bonds.

As can be gleaned from Figure 6c, the situation is quite different in the case of [C_7_OHmim]­[FSI]. In this case, networks indicated by black arrows are not spaced by a thick tail domain because alcohol groups can reach across and bridge them or can connect with the same network that their cationic head is part of. These two behaviors are depicted with an inset on the bottom left part of Figure 6c. It is in the comparison between the insets in Figures 6a and 6c that we find the simplest explanation for the vanishing of intermediate range order in the HFILs and with it the prepeak in *S*(*q*); these are just some of the tethering modes available to HFILs that are not accessible to prototypical ILs. It is now easier to understand why these systems display higher viscosity and lower conductivity; cations in HFILs are multiply connected in a variety of ways via Coulomb or hydrogen bond links. For example, Figure 6d shows a different type of arrangement reminiscent of charge alternation in panel b but in this case involving hydrogen bonds.

In conclusion, a small atomic modification in the terminal tail group of ILs, which transforms them into HFILs, results in a dramatic change in the role of cationic tails. For prototypical ILs, these are separators or spacers of charge networks, but in HFILs, they become physical cross-linkers. Of course, physical cross-links are neither as durable nor as strong as chemical cross-links, but in a way, they cause some similar outcomes including lowering conductivity and increasing viscosity. Besides this “velcro”-like effect tethering different charge networks and significantly shortening their separation, OH groups contribute to a variety of new interactions, including alcohol–alcohol and alcohol–ion–alcohol motifs. In particular, the alcohol–alcohol interactions are interesting, as they connect ions of the same charge.

## Supplementary Material





## Notes

ASCII files with topology information that can be used to simulate the systems using the software GROMACS (ZIP)
Methods for the synthesis of HFILs and for the physical measurement of water content, ionic conductivities, densities, viscosities, differential scanning calorimetry (DSC), computational methods, DSC curves, figure with the partial subcomponents of *S*(*q*) for ${{\mathrm{NTf}}_{2}}^{-}$ -based liquids, figure depicting selected pair distribution functions, and tables with experimental densities and with fit parameters for the temperature dependent densities (PDF)
