# Supplementary material for: Tethered from the Head and from the Tail: The Structure of Hydroxyl-Functionalized Ionic Liquids
Source: J Phys Chem Lett. 2025 Dec 11;16(51):13025–31. doi: 10.1021/acs.jpclett.5c03046 (PMC12746441; doi:10.1021/acs.jpclett.5c03046)
Supplement: Supplementary file 2 [file jz5c03046_si_002.pdf]

Supporting Information for: Tethered From the Head and From the Tail; the Structure of Hydroxyl-Functionalized Ionic Liquids

Raphael Ogbodo,† Ieesha Ansar,‡ Clinton Adu,¶ Sharon I. Lall-Ramnarine,*,‡ James F. Wishart,*,§ Andrew J. Nieuwkoop,*,¶ and Claudio J. Margulis*,†

†*Department of Chemistry, The University of Iowa, Iowa City, IA 52242, United States*

‡*Department of Chemistry, Queensborough Community College-CUNY, Bayside, New York 11364, United States*

¶*Department of Chemistry and Chemical Biology, Rutgers University, Piscataway, New Jersey 08854, United States*

§*Chemistry Division, Brookhaven National Laboratory, Upton, New York 11973-5000, United States*

E-mail: slallramnarine@qcc.cuny.edu; wishart@bnl.gov; an567@chem.rutgers.edu; claudio-margulis@uiowa.edu

S.1 Experimental and Computational Methods

Section S.1.1 describes the synthesis of compounds, Section S.1.2 the techniques used to for physical measurements, and Section S.1.3 describes computational methodology.

S.1.1 Synthesis of HFILs

All chemicals were of reagent grade, obtained from commercial sources, and used as received. 1-methylimidazole, 3-bromo-1-propanol and 7-bromo-1-heptanol were purchased from Arctom Scientific. Lithium bis(trifluoromethylsulfonyl)imide, Lithium bis(fluorosulfonyl)imide, and Potassium bis(fluorosulfonyl)imide were obtained from IoLiTec Inc. Dimethyl sulfoxide-d6 was purchased from Cambridge Isotope Laboratories. NMR spectra were obtained using a Bruker 400 MHz spectrometer and analyzed using Bruker Topspin software.

Solvent-free synthesis of 1-hydroxypropyl-3-methylimidazolium bromide [C_3OHmim][Br]

1-Methylimidazole (9.7 mL, 10.0 g, 0.122 mol) was added to a 3-neck round bottom flask, followed by the addition of 3-bromo-1-propanol (11.63 mL, 17.8 g, 0.128 mol) while purging with argon in a dry box. The reaction mixture was then placed in a fume hood and purged with argon for an additional 10 minutes. The reaction mixture was allowed to stir at room temperature for 7 to 8 days. The product was then dried in a vacuum oven at 60 °C for 2 days. A golden-yellow liquid was obtained (26.48 g, 98% yield Molar mass: 221.12 g/mol). ^{1}H (400 MHz; DMSO-d6) δ, ppm 1.91-1.97 (2H, quin), 3.39-3.43 (2H, q), 3.86 (3H, s), 4.22-4.26 (2H, t), 4.71 (1H, s), 7.73 (1H, s), 7.80 (1H, s), 9.20 (1H, s). ^{13}C (100 MHz; DMSO-d6) δ 32.29, 35.72, 46.18, 56.97, 122.35, 123.51, 136.65.

Synthesis of 1-hydroxypropyl-3-methylimidazolium bis(trifluoromethylsulfonyl)imide ionic liquid [C_3OHmim][NTf_2]:

[C_3OHmim][Br] (13.62 g, 0.062 mol) was dissolved in 25 mL of deionized H_2O in a 500 mL round bottom flask. [Li][NTf_2] (17.68 g, 0.062 mol) was dissolved in 30 mL of deionized water and slowly added to the flask. The sample was stirred for 24 hours. The organic phase was then washed with 300 mL (20 x 15 mL) deionized water until an aliquot of the wash showed a negative result for bromide with 2 drops of 50 mM aqueous silver nitrate. The solvent was removed by rotary evaporation and the sample was further dried in a vacuum oven at 60 °C for 9 days to obtain yellow ionic liquid (11.82 g, 45% Molar mass: 421.39 g/mol). ^{1}H (400 MHz; DMSO-d6) δ, ppm 1.90-1.96 (2H, quin), 3.37-3.42 (2H, q), 3.83 (3H, s), 4.20-4.23 (2H, t), 4.70 (1H, s), 7.68 (1H, s), 7.75 (1H, s), 9.09 (1H, s). ^{13}C (100 MHz; DMSO-d6) δ 32.27, 35.69, 57.03, 117.85,121.05, 122.37, 123.53, 136.62.

Synthesis of 1-hydroxypropyl-3-methylimidazolium bis(fluorosulfonyl) imide ionic liquid [C_3OHmim][FSI]:

[C_3OHmim][Br] (26.58 g, 0.120 mol) was dissolved in 25 mL of deionized water in a 500 mL round bottom flask. Separately, [K][FSI] (26.29 g, 0.120 mol) was dissolved in 30 mL of deionized water and then slowly added to the reaction flask. The mixture was stirred for 48 hours and then rotary evaporated. The white precipitate observed was removed by filtering three times using 60 mL of dichloromethane and 50 mL of ethanol for each filtration. The sample was then rotary evaporated, redissolved in 20 mL of dichloromethane and washed with 440 mL (22 x 20 mL) of deionized water until an aliquot of the wash showed a negative result for bromide when tested with 2 drops of 50 mM aqueous silver nitrate. The solvent was removed by rotary evaporation and the product was dried in a vacuum oven at 60 °C for 10 days, resulting in a yellow ionic liquid (11.83 g, 31%). ^{1}H (400 MHz; DMSO-d6) δ, ppm 1.90-1.97 (2H, quin), 3.40-3.47 (2H, q), 3.84 (3H, s), 4.20-4.24 (2H, t), 4.70 (1H, s), 7.69 (1H, s), 7.75 (1H, s), 9.09 (1H, s). ^{13}C (100 MHz; DMSO-d6) δ 32.76, 36.18, 46.72, 57.53, 122.86, 124.02, 137.12. ^{19}F (376 MHz; DMSO-d6) δ 53.20.

Solvent-free synthesis of 1-hydroxyheptyl-3-methylimidazolium bromide [C_7OHmim][Br]

1-Methylimidazole (10.0 g, 0.122 mol) was added to a 3-neck round bottom flask, followed by the addition of 7-bromo-1-heptanol (24.97 g, 0.128 mol) while purging with argon in a dry box. The reaction flask was then placed in a fume hood, fitted with a condenser, and purged with argon for an additional 10 minutes. The reaction mixture was heated in an oil bath at 55 °C and left to stir for 12 days. The reaction mixture was then rotary evaporated and dried a vacuum oven at 60 °C for 6 days. The final product was a dark brown liquid (34.48 g, 99% Molar mass: 277.20 g/mol). ^{1}H (400 MHz; DMSO-d6) δ, ppm 1.19-1.28 (7H, m), 1.36-1.43 (2H, quin), 1.74-1.81 (2H, quin), 3.35-3.38 (2H, t), 3.86 (3H, s) 4.15-4.19 (2H, t), 7.73 (1H, s), 7.80 (1H, s), 9.19 (1H, s). ^{13}C (100 MHz; DMSO-d6) δ 25.27, 25.50, 28.23, 29.33, 32.33, 35.73, 48.72, 60.57, 122.23, 123.58, 136.45.

Synthesis of 1-hydroxyheptyl-3-methylimidazolium bis (trifluoromethyl sulfonyl) imide ionic liquid [C_7OHmim][NTf_2]:

[C_7OHmim][Br] (18 g, 0.065 mol) was dissolved in 25 mL of deionized water in a 500 mL round bottom flask. [Li][NTf_2] (18.64 g, 0.065 mol) was then dissolved in 30 mL of deionized water and slowly added to the flask. The mixture was stirred for 24 hours. The organic phase was washed with 250 mL (25 x 10 mL) of deionized water until an aliquot of the wash tested negative for bromide using 2 drops of 50 mM aqueous silver nitrate. The dark brown liquid product was dissolved in 30 mL of ethyl acetate and stirred with 1.8 g (0.06 mass equivalent) of activated charcoal for 48 hours. The sample was then filtered using Whatman ashless no. 42 filter paper on a Buchner funnel while rinsing with ethanol, followed by filtering with a fine sintered glass frit. The solvent was removed by rotary evaporation and the product was dried in a vacuum oven at 60 °C for 7 days to yield a yellow ionic liquid (27.21 g, 88% Molar mass: 477.47 g/mol). ^{1}H (400 MHz; DMSO-d6) δ, ppm 1.22-1.29 (6H, q), 1.37-1.43 (2H, quin), 1.74-1.81 (2H, quin), 3.36-3.39 (2H, t), 3.86 (3H, s) 4.13-4.17 (2H, t), 4.32(1H, s), 7.70 (1H, s), 7.76 (1H, s), 9.10 (1H, s). ^{13}C (100 MHz; DMSO-d6) δ 25.75, 25.99, 28.72, 29.81, 32.83, 36.20, 49.24, 61.08, 118.35, 121.55, 122.73, 124.08, 136.95.

Synthesis of 1-hydroxyheptyl-3-methylimidazolium bis(fluorosulfonyl) imide ionic liquid [C_7OHmim][FSI]:

[C_7OHmim][Br] (16.69 g, 0.060 mol) was dissolved in 25 mL of deionized water in a 500 mL round bottom flask. [K][FSI] (13.15 g, 0.060 mol) was then dissolved in 30 mL of deionized water and slowly added to the flask. The mixture was stirred for 24 hours. Following this, the organic phase was washed with 275 mL (25 x 11 mL) of deionized water until an aliquot of the wash tested negative for bromide using two drops of 50 mM aqueous silver nitrate. The dark brown liquid product was dissolved in 30 mL of ethyl acetate and stirred with 1.5 g (0.06 mass equivalents) of activated charcoal for 48 hours. The sample was then filtered using Whatman ashless no. 42 filter paper on a Buchner funnel while rinsing with ethanol followed by filtering using a fine sintered glass frit. The solvent was removed by rotary evaporation and the product was further dried in a vacuum oven at 55 °C for 7 days to yield a yellow ionic liquid (17.34 g, 77% Molar mass: 377.45 g/mol). ^{1}H (400 MHz; DMSO-d6) δ, ppm 1.22-1.29 (6H, q), 1.37-1.43 (2H, quin), 1.74-1.81 (2H, quin), 3.36-3.39 (2H, t), 3.85 (3H, s) 4.13-4.17 (2H, t), 4.33(1H, s), 7.69 (1H, s), 7.76 (1H, s), 9.10 (1H, s). ^{13}C (100 MHz; DMSO-d6) δ 25.76, 26.00, 28.72, 29.82, 32.83, 36.22, 49.24, 61.09, 122.73, 124.08, 136.95. ^{19}F (376 MHz; DMSO-d6) δ 53.20.

S.1.2 Physical Measurements

Water Content

Water contents of the ionic liquid samples were determined using a Mettler Toledo DL39 coulometric Karl Fischer titrator connected to an analytical balance. The water contents of the samples used for the physical property measurements ranged from 22-249 ppm.

Ionic conductivities

Conductivity data was obtained using a YSI 3253 conductivity probe with a YSI 3200 meter. The measurements were performed at room temperature in a dry compressed-air-purged dry box when

the moisture content was less than 1%.

Densities

The density of the ionic liquids were measured using a 2 mL pycnometer. The pycnometer was calibrated with deionized water at room temperature to determine the volume. The pycnometer was then filled with the ionic liquid and placed in a vacuum oven set to the desired temperature, without closing the vacuum. After thermal equilibration, the pycnometer was weighed on an analytical balance, and the density was calculated from the measured mass and calibrated volume.

Differential Scanning Calorimetry

The DSC measurements were done with the TA Instruments DSC Q200 instrument, combined with the Refrigerated Cooling System (RCS-90), which operates in a temperature range from 183.15 K to 823.15 K. The setup runs on the Advantage software v5.5.24 on the Windows 10 operating system. Experimental data were evaluated using the TA universal analysis software. To maintain a dry and inert atmosphere, the analyses were run with a constant dry nitrogen flow inside the furnace. Approximately 10-20 mg of sample was added to a closed aluminum DSC pan. In all cases, the samples were dried by heating for 24 hours under vacuum before each measurement. Each sample was cooled to -90 °C as a starting temperature and heated at 5 °C/min. to 60 °C for the FSI^- and 20 °C for the NTf_2^- salts. The onset glass transition temperatures for the studied ILs are shown in Table 1 in the main manuscript. Figure S.1 through S.4 show the DSC data for the hydroxyl-functionalized ILs.

Viscosities

Viscosities were measured with a Cambridge Applied Systems ViscoLab 4100 electromagnetic reciprocating piston viscometer that was temperature regulated by a Lauda RM-6 circulating bath with a 70/30 v/v propylene glycol/water mixture. The viscometer was calibrated with S600S viscosity reference standard from Koehler Instrument Company (Bohemia, NY). The viscometer was

housed in a moisture controlled dry box with an active dry compressed air purge during data acquisition. The dry box was fitted with a hygrometer and measurements were made when the moisture level was less than 2%. Viscosities for each IL were recorded at intervals between 1 °C and 95 °C in ascending and descending order within their liquid ranges. The data were then fit using the logarithmic form of the Vogel-Tammann-Fulcher relation,

$$\ln\big(\eta(T)\big) = \ln(\eta_0) + \frac{DT_0}{T - T_0}, \tag{S.1}$$

in order to obtain better fitting of points in the low-viscosity range. Each data set included a value of 1 x 10^{13} cP at the liquid's glass transition temperature T_g, according to the practice of Angell,[1] which leads to more consistent trends across a set of liquids. Values of density, conductivity, viscosity, $\ln(\eta_0)$, D and T_0 are given in Table 1 in the main manuscript.

S.1.3 Computational Methods

All classical MD simulations were carried out using the GROMACS software package version 5.1.4.[2] The initial configuration of each IL containing 1024 ion pairs was packed using the PACKMOL[3] software and then energy minimized. Three successive equilibration steps were then performed in the NPT ensemble using the V-rescale thermostat[4] and Berendsen barostat[5] with 0.2 and 1.0 ps time constants, respectively. The first step was run for 5 ns at a pressure of 50 bar and scaled partial charges of 1% of their correct values; the duration of the second step was 5 ns and was run at a pressure of 50 bar and scaled partial charges of 10%, whereas the third step was run for 20 ns at a pressure of 1 bar and 100% partial charges. After these preliminary equilibration steps at 300 K, we further subjected the systems to a 45 ns simulated annealing scheme in which the temperature was ramped from 300 K to 700 K and then brought back to a target temperatures of 300 K and 380 K. Final configurations of these annealing runs were used as initial conditions for 20 ns equilibration runs in the constant pressure and temperature (NPT) ensemble followed by final 20 ns production runs. The cut-off for all the non-bonded interactions (Van der Waals and

electrostatic) during equilibration was set to 10 Å and to 15 Å during production. The particle Mesh Ewald[8,9] method was used for the long-range electrostatic interactions with a fourth-order interpolation and Fourier spacing of 1.2 Å. 3D periodic boundary conditions were applied as coded in GROMACS. Equations of motion were integrated using a time step of 1.0 fs with the MD integrator.[10,11] Classical potential parameters for our simulations, which for convenience we provide as GROMACS input files, were derived from a combination of sources including OPLS-AA,[12] the Canongia Lopes & Pádua force fields,[13–16] Price *et al.*,[17] Shimizu *et al.*,[18] and Kaminski *et al.*[19] Bond, angles, and torsion parameters involving tail oxygen atoms were taken from OPLS-AA,[12] Weiner,,[20] and Cornell *et al.*[21]

Annealing and production runs were carried out using the Nose-Hoover thermostat[6] and the Parrinello-Rahman barostat[7] with 0.2 and 1.0 ps time constants, respectively. Structures from the last 2 ns were used for data collection.

S(q) was calculated using the equation

$$\mathrm{S(q)} = \frac{\rho_0 \sum_i \sum_j x_i x_j f_i(q) f_j(q)}{[\sum_i x_i f_i(q)]^2} \int_0^{\frac{L}{2}} 4\pi r^2 \left(g_{ij}(r) - 1\right) \left(\frac{\sin(qr)}{qr}\right) W(r) dr, \tag{S.2}$$

where $g_{ij}(r)$ is the radial distribution function and $W(r)$ is a Lorch-type function[22,23] that minimizes finite box truncation errors. ρ_o is the average total number density over the frames used. Following our previous work,[24–36] we decompose S(q) into additive ionic and sub-ionic contributions to extract structural information across multiple length scales. Subspecies are defined by the color-coded molecular fragments shown in Fig. 1a.

S.2 Additional Tables and Figures

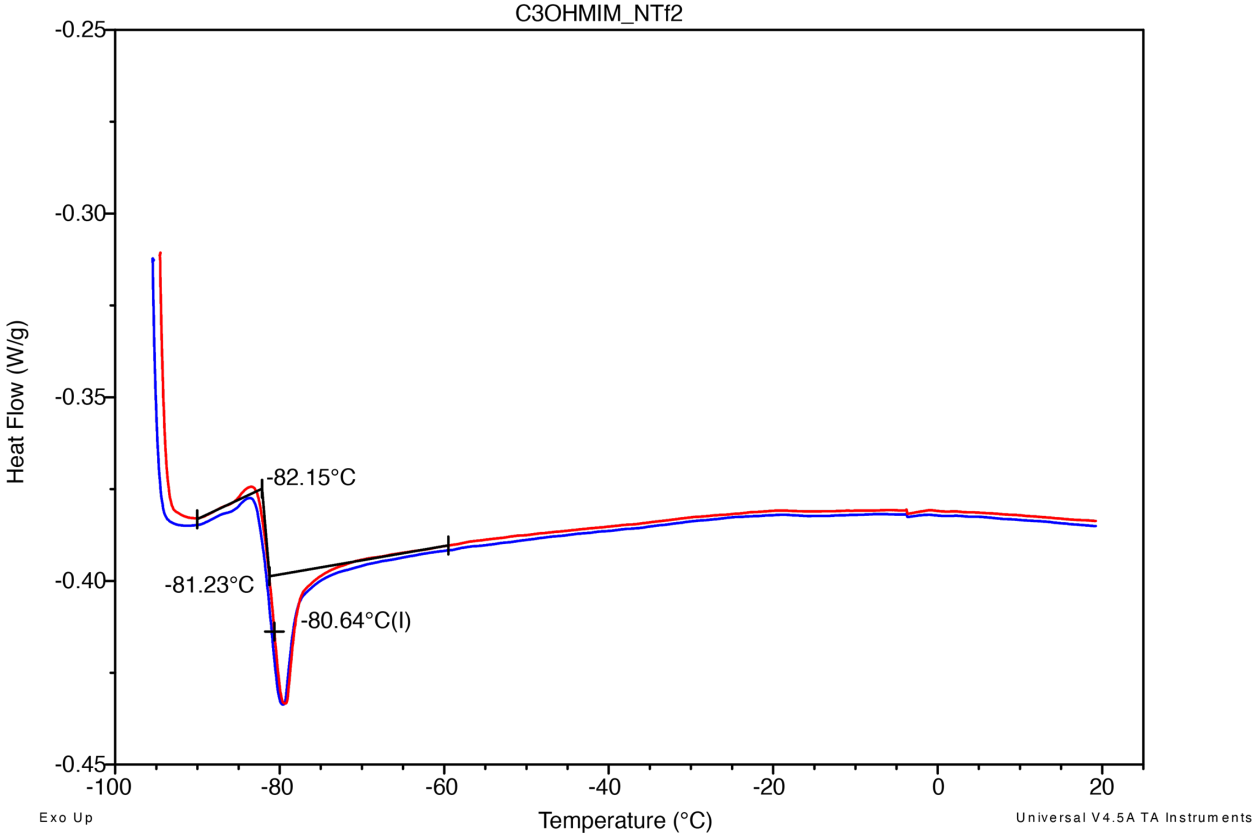

Figure S.1: DSC data for [C_3OHmim][NTf_2].

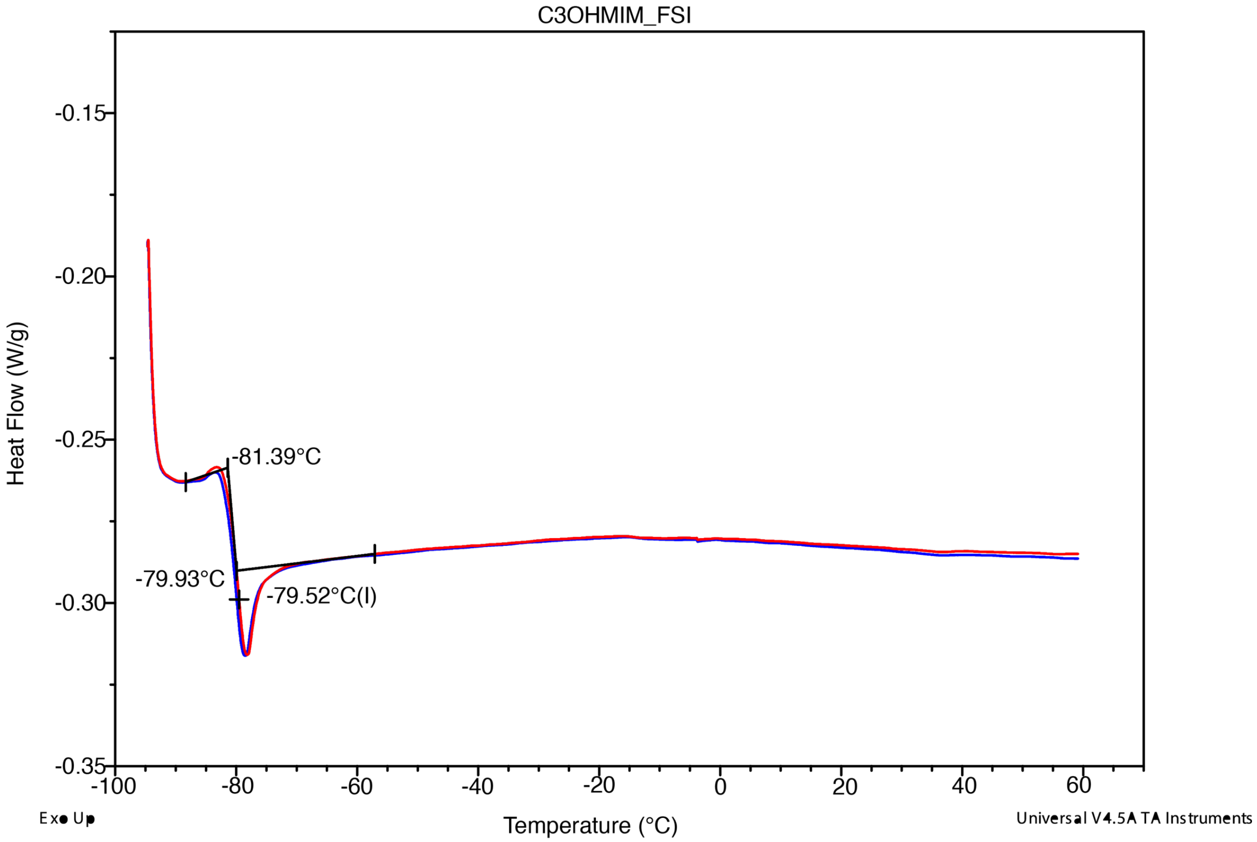

Figure S.2: DSC data for [C_3OHmim][FSI].

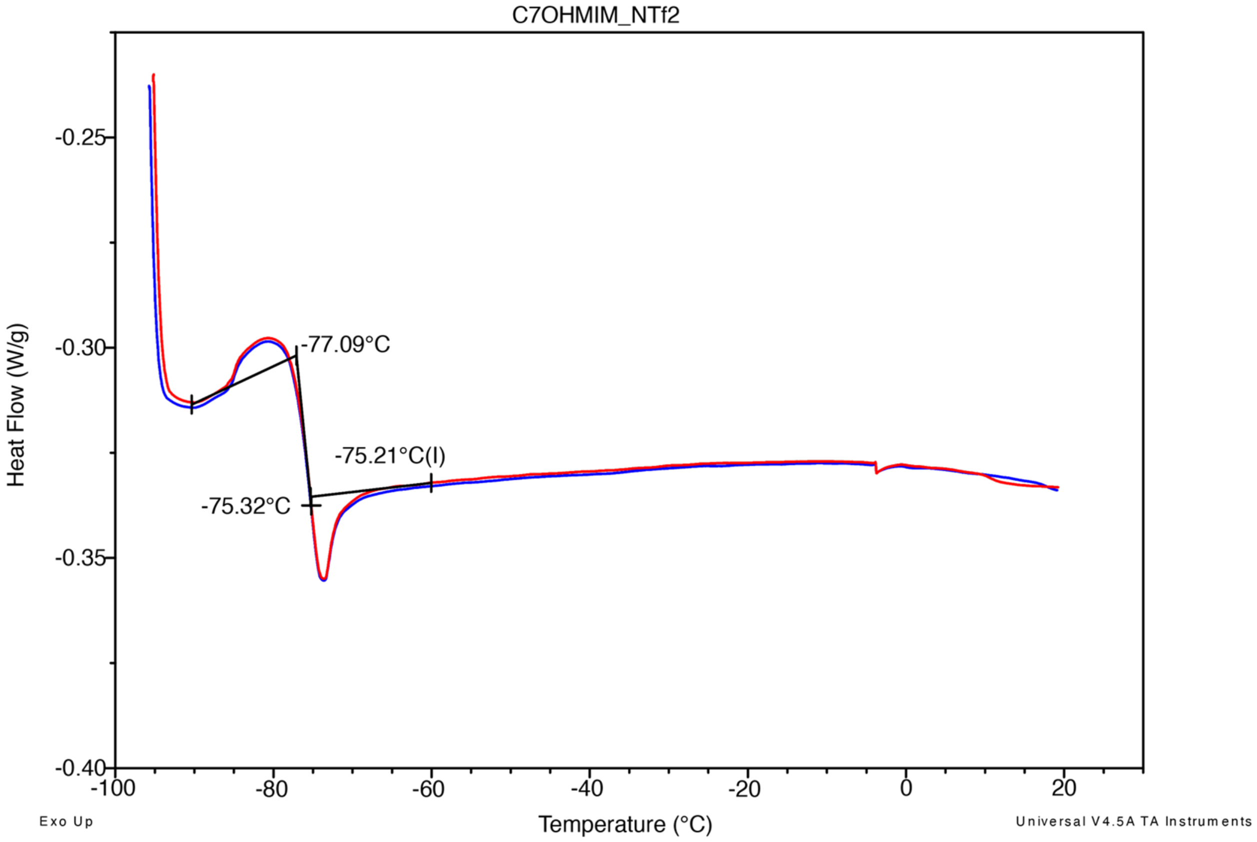

Figure S.3: DSC data for [C_7OHmim][NTf_2].

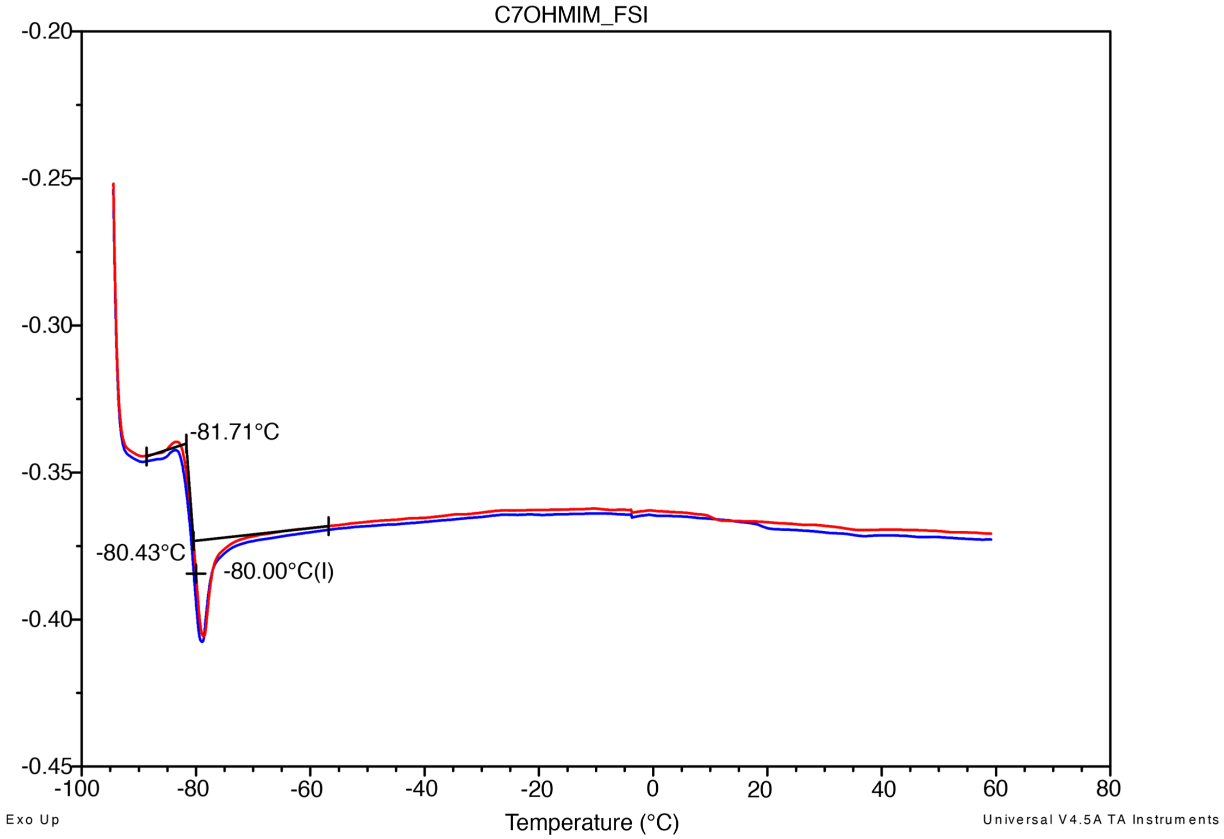

Figure S.4: DSC data for [C_7OHmim][FSI].

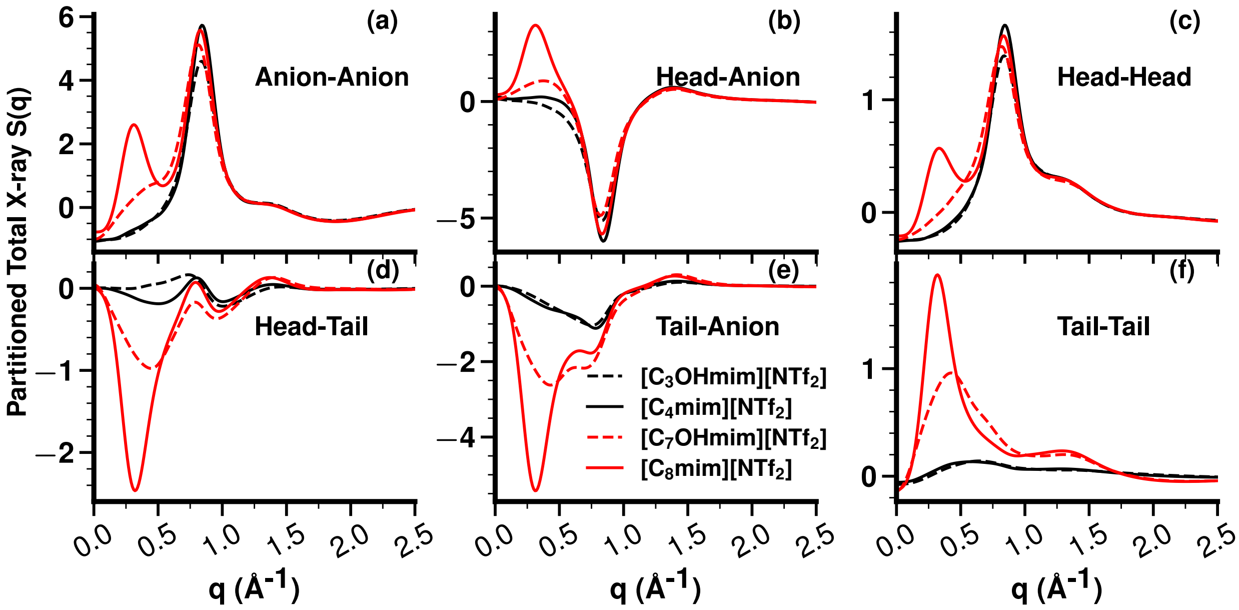

Figure S.5: For C_8mim^+, C_7OHmim^+, C_4mim^+, C_3OHmim^+, all coupled with NTf_2^-; sub-ionic components of the total X-ray S(q). Matching plots in the case of FSI^- are shown in Fig. 4.

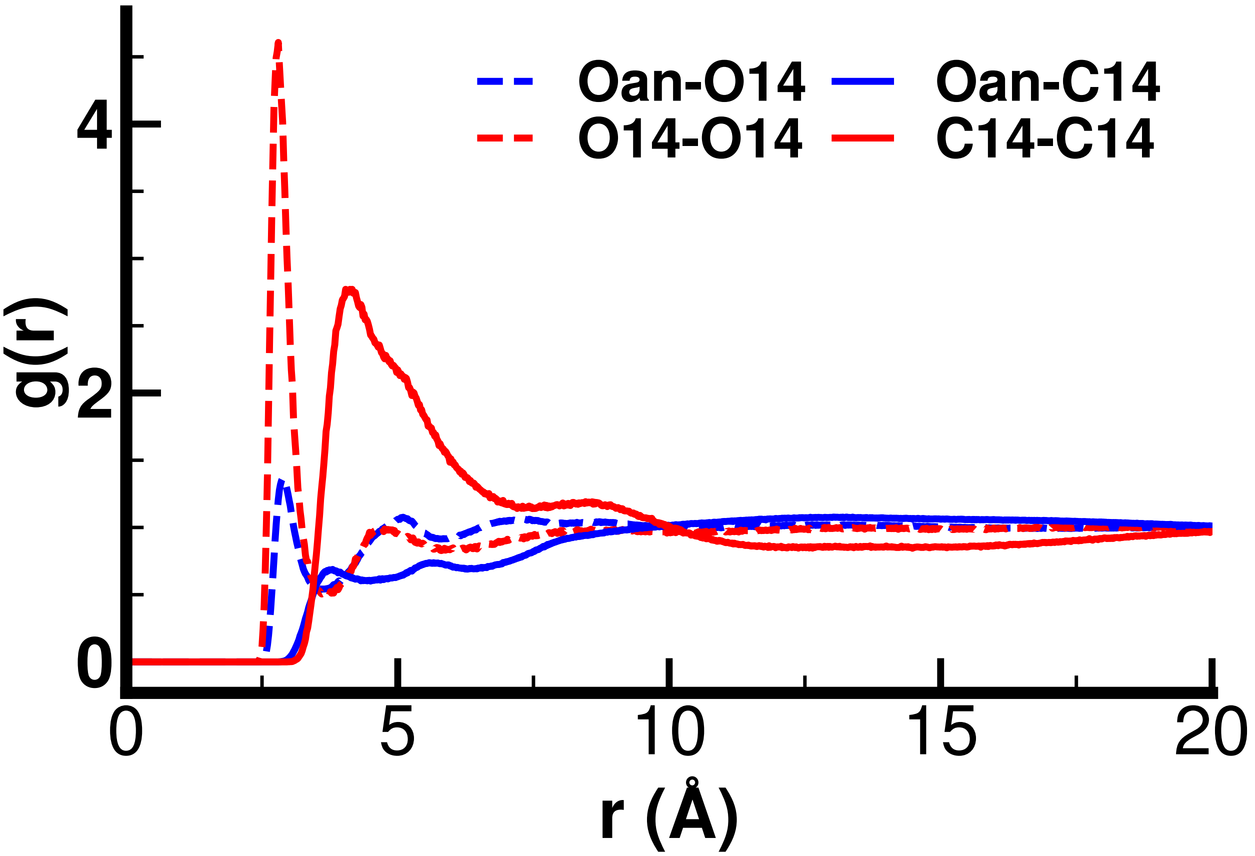

Figure S.6: For [C_7OHmim][FSI] and [C_8mim][FSI], interionic pair distribution functions between Oan (anionic oxygen) and O14, Oan and C14, C14 and C14, and O14 and O14.

Table S.1: Experimental densities ρ (g/mL) as a Function of Temperature.

T (K)	[C_3OHmim][FSI]	[C_3OHmim][NTf_2]	[C_7OHmim][FSI]	[C_7OHmim][NTf_2]	[C_8mim][FSI]
296.15		1.5277			
297.15	1.477				
298.15			1.3184	1.3991	1.2493
308.15		1.5088			
313.15	1.4549				
316.15			1.3091		1.2344
318.15				1.3604	
323.15		1.4997			
327.15	1.4426				1.2236
328.15			1.2945		
338.15				1.3628	1.2128
339.15		1.4983			
343.15	1.4290		1.2847		
353.15	1.4223	1.4820		1.3412	1.2021
358.15			1.2732		

Table S.2: For Liquids in the Left Column, Parameters to Fit the Density $\rho(\mathrm{T}) = \mathrm{c}_\rho \mathrm{T} + \rho_0$, and the Mean Standard Deviation δ, From Their Respective Regression Analyses.

Ionic Liquid	c_ρ (g/mL/K)	ρ_0 (g/mL)	δ
[C_3OHmim][FSI]	-0.00082	1.712	0.00054
[C_3OHmim][NTf_2]	-0.00093	1.812	0.0006
[C_7OHmim][FSI]	-0.00078	1.551	0.0018
[C_7OHmim][NTf_2]	-0.00080	1.633	0.0010
[C_8mim][FSI]	-0.00088	1.510	0.00089

References

(1) Xu, W.; Cooper, E. I.; Angell, C. A. Ionic Liquids:Ion Mobilities, Glass Temperatures, and Fragilities. *J. Phys. Chem. B* **2003**, *107*, 6170–6178.

(2) Hess, B.; Kutzner, C.; Van Der Spoel, D.; Lindahl, E. GROMACS 4: Algorithms for Highly Efficient, Load-Balanced, and Scalable Molecular Simulation. *J. Chem. Theory Comput.* **2008**, *4*, 435–447.

(3) Martínez, L.; Andrade, R.; Birgin, E. G.; Martínez, J. M. PACKMOL: A package for building initial configurations for molecular dynamics simulations. *J. Comput. Chem.* **2009**, *30*, 2157–2164.

(4) Bussi, G.; Donadio, D.; Parrinello, M. Canonical Sampling Through Velocity Rescaling. *J. Chem. Phys.* **2007**, *126*, 014101.

(5) Berendsen, H. J. C.; Postma, J. P. M.; van Gunsteren, W. F.; DiNola, A.; Haak, J. R. Molecular Dynamics with Coupling to an External Bath. *J. Chem. Phys.* **1984**, *81*, 3684–3690.

(6) Nosé, S. A Unified Formulation of the Constant Temperature Molecular Dynamics Methods. *J. Chem. Phys.* **1984**, *81*, 511–519.

(7) Parrinello, M.; Rahman, A. Polymorphic Transitions in Single Crystals: A New Molecular Dynamics Method. *J. Appl. Phys.* **1981**, *52*, 7182–7190.

(8) Darden, T.; York, D.; Pedersen, L. Particle Mesh Ewald: An $N \cdot \log(N)$ Method for Ewald Sums in Large Systems. *J. Chem. Phys.* **1993**, *98*, 10089–10092.

(9) Essmann, U.; Perera, L.; Berkowitz, M. L.; Darden, T.; Lee, H.; Pedersen, L. G. A Smooth Particle Mesh Ewald Method. *J. Chem. Phys.* **1995**, *103*, 8577–8593.

(10) Hockney, R.; Goel, S.; Eastwood, J. Quiet high-resolution computer models of a plasma. *J. Comput. Phys.* **1974**, *14*, 148–158.

(11) González, M. Force fields and molecular dynamics simulations. *JDN* **2011**, *12*, 169–200.

(12) Jorgensen, W. L.; Maxwell, D. S.; Tirado-Rives, J. Development and Testing of the OPLS All-Atom Force Field on Conformational Energetics and Properties of Organic Liquids. *J. Am. Chem. Soc.* **1996**, *118*, 11225–11236.

(13) Canongia Lopes, J. N.; Deschamps, J.; Pádua, A. A. H. Modeling Ionic Liquids Using a Systematic All-Atom Force Field. *J. Phys. Chem. B* **2004**, *108*, 2038–2047.

(14) Canongia Lopes, J. N.; Deschamps, J.; Pádua, A. A. H. Modeling Ionic Liquids Using a Systematic All-Atom Force Field. *J. Phys. Chem. B* **2004**, *108*, 11250–11250.

(15) Canongia Lopes, J. N.; Pádua, A. A. H. Molecular Force Field for Ionic Liquids Composed of Triflate or Bistriflylimide Anions. *J. Phys. Chem. B* **2004**, *108*, 16893–16898.

(16) Canongia Lopes, J. N.; Pádua, A. A. H.; Shimizu, K. Molecular Force Field for Ionic Liquids IV: Trialkylimidazolium and Alkoxycarbonyl-Imidazolium Cations; Alkylsulfonate and Alkylsulfate Anions. *J. Phys. Chem. B* **2008**, *112*, 5039–5046.

(17) Price, M. L. P.; Ostrovsky, D.; Jorgensen, W. L. Gas-phase and Liquid-State Properties of Esters, Nitriles, and Nitro Compounds with the OPLS-AA Force Field. *J. Comput. Chem.* **2001**, *22*, 1340–1352.

(18) Shimizu, K.; Almantariotis, D.; Gomes, M. F. C.; Pádua, A. A. H.; Canongia Lopes, J. N. Molecular Force Field for Ionic Liquids V: Hydroxyethylimidazolium, Dimethoxy-2-

Methylimidazolium, and Fluoroalkylimidazolium Cations and Bis(Fluorosulfonyl)Amide, Perfluoroalkanesulfonylamide, and Fluoroalkylfluorophosphate Anions. *J. Phys. Chem. B* **2010**, *114*, 3592–3600.

(19) Kaminski, G. A.; Friesner, R. A.; Tirado-Rives, J.; Jorgensen, W. L. Evaluation and Reparametrization of the OPLS-AA Force Field for Proteins via Comparison with Accurate Quantum Chemical Calculations on Peptides. *J. Phys. Chem. B* **2001**, *105*, 6474–6487.

(20) Weiner, S. J.; Kollman, P. A.; Nguyen, D. T.; Case, D. A. An All Atom Force Field for Simulations of Proteins and Nucleic Acids. *J. Comput. Chem.* **1986**, *7*, 230–252.

(21) Cornell, W. D.; Cieplak, P.; Bayly, C. I.; Gould, I. R.; Merz, K. M.; Ferguson, D. M.; Spellmeyer, D. C.; Fox, T.; Caldwell, J. W.; Kollman, P. A. A Second Generation Force Field for the Simulation of Proteins, Nucleic Acids, and Organic Molecules. *J. Am. Chem. Soc.* **1995**, *117*, 5179–5197.

(22) Lorch, E. Neutron Diffraction by Germania, Silica and Radiation-Damaged Silica Glasses. *J. Phys. C: Solid State Phys.* **1969**, *2*, 229–237.

(23) Du, J.; Benmore, C. J.; Corrales, R.; Hart, R. T.; Richard Weber, J. K. A Molecular Dynamics Simulation Interpretation of Neutron and X-Ray Diffraction Measurements on Single Phase Y_2O_3-Al_2O_3 Glasses. *J. Phys.: Condens. Matter* **2009**, *21*, 205102.

(24) Araque, J. C.; Hettige, J. J.; Margulis, C. J. Modern Room Temperature Ionic Liquids, a Simple Guide to Understanding Their Structure and How It May Relate to Dynamics. *J. Phys. Chem. B* **2015**, *119*, 12727–12740.

(25) Kashyap, H. K.; Margulis, C. J. (Keynote) Theoretical Deconstruction of the X-ray Structure Function Exposes Polarity Alternations in Room Temperature Ionic Liquids. *ECS Trans.* **2013**, *50*, 301–307.

(26) Hettige, J. J.; Kashyap, H. K.; Annapureddy, H. V.; Margulis, C. J. Anions, the Reporters of Structure in Ionic Liquids. *J. Phys. Chem. Lett.* **2013**, *4*, 105–10.

(27) Araque, J. C.; Hettige, J. J.; Margulis, C. J. Ionic liquids—Conventional Solvent Mixtures, Structurally Different but Dynamically Similar. *J. Chem. Phys.* **2015**, *143*, 134505.

(28) Hettige, J. J.; Araque, J. C.; Kashyap, H. K.; Margulis, C. J. Communication: Nanoscale Structure of tetradecyltrihexylphosphonium based Ionic Liquids. *J. Chem. Phys.* **2016**, *144*, 121102.

(29) Daly, R. P.; Araque, J. C.; Margulis, C. J. Communication: Stiff and Soft Nano-Environments and the "Octopus Effect" are the Crux of Ionic Liquid Structural and Dynamical Heterogeneity. *J. Chem. Phys.* **2017**, *147*, 061102.

(30) Dhungana, K. B.; Margulis, C. J. Comparison of the Structural Response to Pressure of Ionic Liquids with Ether and Alkyl Functionalities. *J. Phys. Chem. B* **2017**, *121*, 6890–6897.

(31) Araque, J. C.; Margulis, C. J. In an Ionic Liquid, High Local Friction is Determined by the Proximity to the Charge Network. *J. Chem. Phys.* **2018**, *149*, 144503.

(32) Amith, W. D.; Araque, J. C.; Margulis, C. J. A Pictorial View of Viscosity in Ionic Liquids and the Link to Nanostructural Heterogeneity. *J. Phys. Chem. Lett.* **2020**, *11*, 2062–2066.

(33) Amith, W. D.; Araque, J. C.; Margulis, C. J. Relationship between the Relaxation of Ionic Liquid Structural Motifs and That of the Shear Viscosity. *J. Phys. Chem. B* **2021**, *125*, 6264–6271.

(34) Sharma, S.; Ivanov, A. S.; Margulis, C. J. A Brief Guide to the Structure of High-Temperature Molten Salts and Key Aspects Making Them Different from Their Low-Temperature Relatives, the Ionic Liquids. *J. Phys. Chem. B* **2021**, *125*, 6359–6372.

(35) Ogbodo, R.; Karunaratne, W. V.; Acharya, G. R.; Emerson, M. S.; Mughal, M.; Yuen, H. M.; Zmich, N.; Nembhard, S.; Wang, F.; Shirota, H. et al. Structural Origins of Viscosity in

Imidazolium and Pyrrolidinium Ionic Liquids Coupled with the NTf_2^- Anion. *J. Phys. Chem. B* **2023**, *127*, 6342–6353.

(36) Borah, B.; Acharya, G. R.; Grajeda, D.; Emerson, M. S.; Harris, M. A.; Milinda Abeykoon, A.; Sangoro, J.; Baker, G. A.; Nieuwkoop, A. J.; Margulis, C. J. Do Ionic Liquids Slow Down in Stages? *J. Am. Chem. Soc.* **2023**, *145*, 25518–25522.
